# The *relBE2Spn* Toxin-Antitoxin System of *Streptococcus pneumoniae*: Role in Antibiotic Tolerance and Functional Conservation in Clinical Isolates

**DOI:** 10.1371/journal.pone.0011289

**Published:** 2010-06-23

**Authors:** Concha Nieto, Ewa Sadowy, Adela G. de la Campa, Waleria Hryniewicz, Manuel Espinosa

**Affiliations:** 1 Centro de Investigaciones Biológicas, Consejo Superior de Investigaciones Científicas, Madrid, Spain; 2 National Medicines Institute, Warsaw, Poland; 3 Centro Nacional de Microbiología and CIBER Enfermedades Respiratorias, Instituto de Salud Carlos III, Majadahonda, Spain; Cairo University, Egypt

## Abstract

Type II (proteic) chromosomal toxin-antitoxin systems (TAS) are widespread in Bacteria and Archaea but their precise function is known only for a limited number of them. Out of the many TAS described, the *relBE* family is one of the most abundant, being present in the three first sequenced strains of *Streptococcus pneumoniae* (D39, TIGR4 and R6). To address the function of the pneumococcal *relBE2Spn* TAS in the bacterial physiology, we have compared the response of the R6-*relBE2Spn* wild type strain with that of an isogenic derivative, Δ*relB2Spn* under different stress conditions such as carbon and amino acid starvation and antibiotic exposure. Differences on viability between the wild type and mutant strains were found only when treatment directly impaired protein synthesis. As a criterion for the permanence of this locus in a variety of clinical strains, we checked whether the *relBE2Spn* locus was conserved in around 100 pneumococcal strains, including clinical isolates and strains with known genomes. All strains, although having various types of polymorphisms at the vicinity of the TA region, contained a functional *relBE2Spn* locus and the type of its structure correlated with the multilocus sequence type. Functionality of this TAS was maintained even in cases where severe rearrangements around the *relBE2Spn* region were found. We conclude that even though the *relBE2Spn* TAS is not essential for pneumococcus, it may provide additional advantages to the bacteria for colonization and/or infection.

## Introduction

Chromosomally-encoded Type II toxin-antitoxin systems (TAS), composed of two proteins, are widely spread among Bacteria and Archaea. Typically, they are organized as operons in which the first gene encodes the antitoxin (A) and the second the toxin (T). Both proteins interact to generate a harmless TA complex that auto-regulate their own synthesis. The A protein by itself is metabolically unstable and is constitutively degraded by ATP-dependent proteases, releasing a free and stable T protein that would kill or stop the growth of the cells by disruption of key cellular processes [1]. A puzzling observation derived from bio-informatics approaches is that many bacteria and archaea harbour multiple copies of various TAS (e.g. around 60 TAS in *Mycobacterium tuberculosis* [2]), being even more abundant than previously envisaged [3], [4]. Notwithstanding the knowledge on the mechanisms of action of TAS [5] and the three-dimensional structure of various TA protein complexes [6]–[12], little is known on the role of these systems in the bacterial cell lifestyle. In the case of plasmid-encoded TAS, they seem to be involved in the stable maintenance (“addiction”) of the replicons by increasing their chances of vertical transmission [13]. For the chromosomally-encoded TAS, several interpretations have been given to their ubiquity and abundance, though none has been demonstrated thus far [14]. First, it has been proposed that TAS could act as stress response elements that modulate growth by reducing macromolecular synthesis. Hence, induction of these systems results in cell stasis rather than in cell death, leading to viable but not cultivable cells [5], [15]. Inhibition of bacterial growth induced by the toxin was reversed by expression of the cognate antitoxin or by the transfer-messenger mRNA (tmRNA). Thus, toxins would induce a reversible stasis that improves bacterial cell survival under extreme conditions [15]–[17]. Second, some chromosomal TAS such as *mazEF* has been considered as mediators of bacterial programmed cell death [18], [19]. Unfavourable cell growth conditions could trigger this pathway and, as a consequence, a subpopulation of bacterial cells would die. Death of these cells would i) preserve the food for the remaining population, ii) serve as a defence mechanism to restrict phage spreading, and iii) act as a mechanism to eliminate cells with deleterious mutations. It would seem that *mazEF*-mediated cell death is a population-dependent phenomenon requiring a quorum sensing molecule, termed extracellular death factor, which is a linear pentapeptide (NNWNN) important for *mazEF*-mediating killing activity [20]. *E. coli* strains defective in *mazEF* showed lower sensitivity to antibiotics than the wild type, indicating that antibiotic addition could induce *mazEF*-mediated cell death [21]. And third, comparison of the fitness of two isogenic *E. coli* strains, one wild type (wt) and the other having deletions in five TAS (*mazEF*, *relBE*, *chpBK*, *yefM-yoeB*, *dinJ-yafQ*) subjected to short-term stress conditions (amino acid starvation, acidic stress, antibiotic treatment, and long-term stationary phase) showed no significant differences among them [22], pointing that TAS could be involved only in long-term evolution [1]. However, some findings have complicated further the interpretation of the TAS role: i) TAS-defective cells showed a reduced ability for biofilm formation [23], [24]; ii) TA-cassettes have been localized in both integrative and conjugative genome elements that could have incorporated into the bacterial chromosome and, within this context, they could promote plasmid maintenance [2], [25]–[28]; iii) TAS can work as anti-addiction modules [29]; iv) they may play an essential role in development of programmed cell death which leads to *Myxococcus* multicellular development [30], and v) they may be linked to bacterial persistence upon antibiotic exposure [31].

Genes for at least eight putative TAS are present in the chromosome of the Gram-positive bacterium *Streptococcus pneumoniae* (the pneumococcus): *relBE1Spn, relBE2Spn*, *yefMyoeBSpn*, *higAB*, *phd/doc*, *pezAT*, *tasAB*, and *hicAB* [3], [17], [32]. Among these, only three of them, namely *relBE2Spn* [33], *yefM-yoeBSpn* [34], and *pezAT* [7] have been shown to be genuine TAS, whereas *relBE1Spn* was shown to be non-functional [33]. Exposure of *E. coli* cells to RelE2*Spn* toxin resulted in the arrest of cell growth, which was rescued by induction of RelB2*Spn* antitoxin but only within a time-frame window: long-time exposure to the toxin led to cultures unable to resume growth [33]. We report here on the role of the pneumococcal *relBE2Spn* TAS in the bacteria lifestyle. We have compared the behaviour of two pneumococcal isogenic strains, wild type (wt) R6 and a *relBE2Spn* mutant derivative (R6*ΔrelB2Spn*) [33] under different growth conditions, and differences were found when cells were subjected to stress conditions that impaired protein synthesis. The RelE2*Spn* toxin could act as a modulator of protein synthesis under stress, but it could also induce cell death when the level of protein synthesis was dramatically reduced. Further, if *relBE2Spn* played a role in bacterial fitness, then it should contribute to colonization and survival after infection (an important part of the switch from commensal to infective for a bacterium like *S. pneumoniae*). If this was the case, the *relBE2Spn* genes should be ubiquitous in the *S. pneumoniae* population. Thus, the presence and integrity of the *relBE2Spn* locus was tested in 100 strains from different sources. Unlike *E. coli*, where several strains lacked the *relBE* operon [1], [14], all pneumococcal strains analyzed retained this locus in their chromosome. Although the *relBE2Spn* operon exhibited various degrees of polymorphisms in the different isolates, none of the changes impaired the functionality of the *relBE2Spn* locus. A molecular model of the pneumococcal RelE2*Spn* protein was constructed based on the three dimensional structure of the RelBE complexes from *Pyrococcus horikoshii* (*Ph*RelE) [10] and compared with that of *Methanococcus jannaschii* (*Mj*RelE) [11]. The modelled RelE2*Spn* kept several residues related to the catalytic activity of ribonucleases, which are also present in *Mj*RelE. However, these residues are missing in *Eco*RelE and *Ph*RelE proteins [10], [35], [36], raising the possibility that the two former RelE proteins, albeit being ribonucleases, could use a mechanism of action different than the one proposed for *Eco*RelE [12].

## Results

### Mutation of the *relBE2Spn* operon has no effect on pneumococcal cell viability under either regular conditions of growth or carbon starvation

We first tested the differences in growth (optical density at 650 nm, OD_650_) and viability (measured by determination of the colony-forming units, cfu) between the two strains, R6 wt and its *relBE2Spn* mutant derivative, R6*ΔrelB2Spn* (Table 1). The mutant strain contains two mutated copies of the *relB2* gene, and has the *relE2* gene placed away from its promoter, also disrupting the translational coupling that appears to exist in this pneumococcal operon [33]. RT-PCR assays showed that in the mutant strain there was not detectable synthesis of *relE*2 mRNA (not shown). To perform the experiments, cultures of both strains were grown 24 h, and OD_650_ and cfu were determined at time intervals. No differences were found between the strains during the entire period in which the cultures passed through exponential (0–2 hrs), stationary (2–8 hrs), and autolysis (8–24 hrs) phases of growth (Figure S1 A, B). Autolysis is a distinctive property of *S. pneumoniae* whose cells show a tendency to spontaneously lyse when the culture reaches the stationary phase [37]. Autolysis plays an important role in the bacterial infection by the release of virulence factors which may modulate the inflammatory response [38]. Glucose starvation activates *relBE* transcription in *E. coli*, probably because of degradation of RelB by the protease Lon, an event that would lead to an increase in free RelE toxin and a reduction in the number of cfu [39]. In the case of *S. pneumoniae*, the sugars routinely used as a carbon source are di-saccharides (sucrose or maltose) rather than glucose because of poorer utilization of the latter [40]. Carbon-starved cultures of either wt or Δ*relB2Spn* mutant cells showed no differences although cessation of growth was observed for either strain as compared to sucrose-grown cultures, and no decrease in viability was observed in either sucrose-depleted culture (Figure S1 C, D).

**Table 1:** Bacterial strains and plasmids.

Bacteria	Genotype	Reference/source
*E. coli* TOP10	*F-mcrA, Δ(mrr-hsdRMS-mcrBC), Φ80lacZΔM15ΔlacX74, recA1, deoR, araD 139Δ(ara-leu)7697, galU, galK, rpsL(St^R^), endA1, nupG*	Invitrogen
*S. pneumoniae* R6	Wild type	[40]
*S. pneumoniae* R6*ΔrelB2Spn*	R6, *ΔrelB2Spn, Cm^R^*	[33]

Plasmid	Features	Reference/source
pFUS2	pMB1, P*_BAD_*, *araC*, *neo*	[50]
pNM220	mini-R1, pA1/O4/O3, *bla, lacI^q^*	[51]
pE2wt	pFUS2, *relE2Spnwt*	This work
pE71	pFUS2, *relE2Spn7153*	This work
pE81	pFUS2, *relE2Spn8651*	This work
pE600	pFUS2, *relE2SpnK-600*	This work
pEter	pFUS2, *relE2Spn7153ter*	This work
pB2wt	pNM220, *relB2Spnwt*	This work

### The *relBE2Spn* operon modulates pneumococcal growth under amino acid starvation

**Figure 1:**
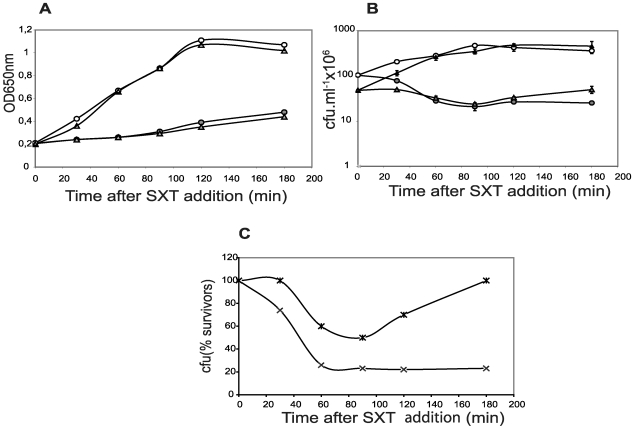
Changes in the growth profile of *S. pneumoniae* cells of the R6 or the R6Δ*relB2Spn* mutant strains after inhibition of protein synthesis by SHT. *S. pneumoniae* cultures, wt (○) and mutant (Δ) cells, were grown exponentially until an OD_650_  = 0.2. Then, SHT (1.5 mg.ml^−1^) was added, and incubation proceeded. Growth was monitored by determination of the OD_650_ of the cultures treated (filled symbols) or untreated (open symbols) with SHT (A). At the times indicated, the numbers of cfu were determined by plating appropriate cell dilutions on SHT-free medium, and incubation for 36 h at 37°C (B). Percentages of viable cells from wt (x) or mutant (*) strains upon SHT-treatment (C) were calculated considering the number of cfu at time zero of the treatment as 100%.

**Figure 2:**
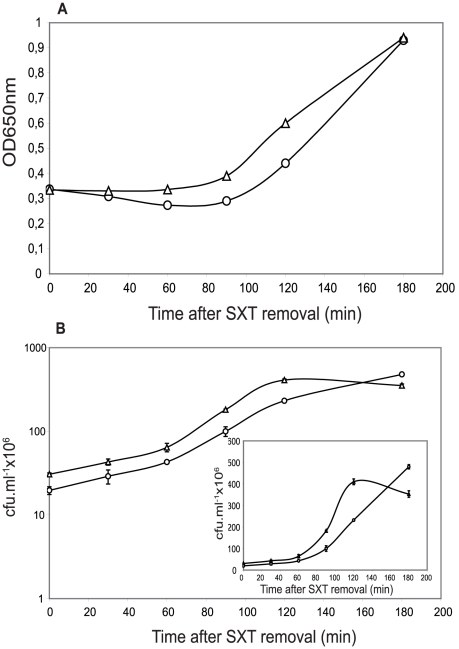
Recovery of cell growth after removal of SHT. SHT-amino acid starved pneumococcal cells (180 minutes of starvation) were washed twice and suspended into pre-warmed fresh medium, and incubation proceeded for the indicated times. Recovery of starvation was followed by turbidity of the cultures (OD_650_) of wt (○) and mutant (Δ) strains (A). The number of cfu was determined by plating appropriate dilutions of the cells at the times indicated (B). **Inset:** Linear plot showing cell viability evolution after SHT removal in wt (○) or mutant (Δ) cultures.

Serine hydroxamate (SHT) induces amino acid starvation because it blocks incorporation of Ser residues into proteins by interfering with the load of seryl-tRNA [41], [42]. In *E. coli*, addition of SHT (similarly to carbon starvation) resulted in an increase of *relBE* transcription leading to the increase of free RelE due to RelB Lon-dependent proteolysis [39]. Thus, we followed growth and viability of the wt and the mutant strains of *S. pneumoniae* under a SHT-mediated amino acid starvation. The results (Figure 1A) showed that the growth of both cultures almost stopped, quickly and in a similar manner, indicating that cells entered into stasis. In contrast to sucrose starvation, differences in viability between both strains were observed (Figure 1B). In the mutant strain, a 50%-reduction in cfu was detected during the first 90 min of SHT treatment, followed by a slight recovery at longer times. Such a recovery was not detected for the wt strain, in which a continuous reduction in cfu was seen; these differences were more evident when the cfu were plotted on a linear, rather than logarithmic scale (Figure 1C). After the 180 min starvation period, SHT was removed, cells were resuspended in fresh pre-warmed medium, and incubation was continued for additional 180 min (Figures 2A and 2B). SHT withdrawal allowed resumption of bacterial growth. However, the mutant cells recovered and entered into stationary phase faster than the wt, whereas the latter showed a more prolonged exponential phase (Figure 2B, inset). These results suggested to us that i) the mutant strain could recover faster because it lacked toxin RelE, and ii) the wt cells could have saved more efficiently their physiological resources during stasis, allowing a full recovery after amino acid starvation.

### Blocking protein synthesis by erythromycin or streptomycin treatment leads to antibiotic tolerance in the *relBE2Spn* mutant

**Figure 3:**
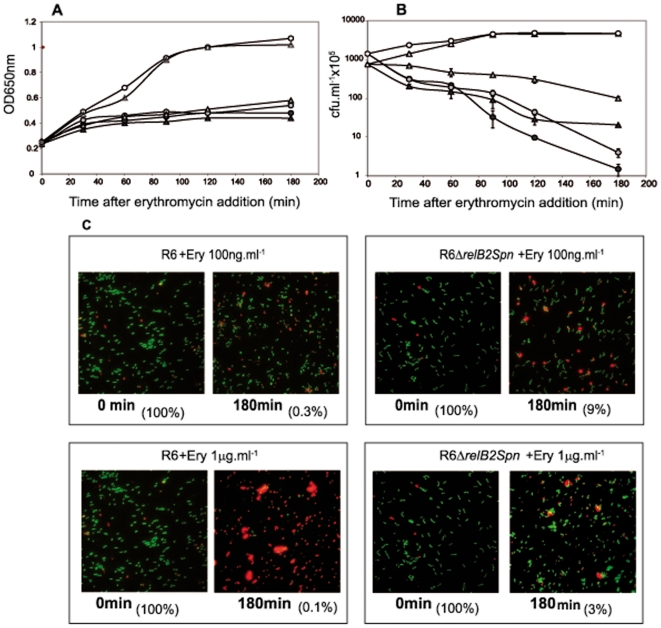
Inhibition of protein synthesis by Erm treatment. Cultures of R6 (○) or R6Δ*relB2Spn* mutant (Δ) cells were treated with two concentrations of Erm, 1 µg.ml^−1^ (black symbols) or 0.1 µg.ml^−1^ (grey symbols). Growth was followed by absorbance at OD_650_ (A) of untreated (open symbols) and treated (filled symbols) cultures. At the indicated times, appropriate dilutions of cells were plated and incubated as above (B) All experiments were performed at least three times. LIVE/DEAD-staining of wt and the R6Δrel*BE2Spn* cells (C). The cultures were harvested 180 min after addition of Erm and stained with Baclight Bacterial viability Kit (Invitrogen). Live cells show green fluorescence, whereas dead cells fluoresce red. Percentages of viable cells in every assay, calculated from the results of Figure 3B are indicated in parentheses.

In addition to carbon- and amino acid-induced starvation, treatment with inhibitors of protein synthesis also caused a *relBE* transcriptional induction in *E. coli* [39]. We employed erythromycin (Erm) which inhibits protein synthesis by binding to the 23S rRNA, interfering with the amino acyl translocation [43]. Erm is an effective agent against streptococcal infections and its minimal inhibitory concentration (MIC) is low, since for selection for pneumococcal Erm-resistant transformants, the concentration 1 µg.ml^−1^ is sufficient [44] and MIC for the majority of wild-type isolates fall in the range of 0.032–0,125 µg.ml^−1^ (http://www.eucast.org/mic_distributions/). To test whether the mutation of *relBESpn* had any effect on cell-response to blocking protein synthesis, pneumococcal cultures (wt and Δ*relB2Spn* strains) were challenged with two dosages of Erm, 0.1 and 1 µg.ml^−1^ (that is 10 and 100 fold MIC for R6, respectively), followed by OD_650_ and cfu determination at different times. After the 20 min treatment, growth of both cultures was stopped, concomitantly with a progressive reduction in cfu (Figures 3A and 3B). At the end of the incubation period (180 min), a 10 to 1000-fold reduction in cfu was observed. Interestingly, the reduction in cfu was much more pronounced (10- to 100-fold difference) in the wt than in the mutant strain. These findings indicate that activation of RelE after antibiotic treatment would induce a complete shut-off in protein synthesis leading to cell stasis or even cell death. Then, lack of the *relBE2Spn* operon would lead to antibiotic tolerance.

To confirm these observations, a test of bacterial viability was performed by employment of the LIVE/DEAD Baclight (Invitrogen) stain method. The pneumococcal cultures were stained and examined under the microscope: living cells were stained in green, whereas dead cells were stained in red. Micrographs of the pneumococcal cultures were taken at 0 and after 180 min of Erm treatment. The results showed no significant differences between both strains when the cultures were challenged with 0.1 µg.ml^−1^ of Erm (Figure 3C upper panel). When Erm concentration was raised to 1 µg.ml^−1^, a drastic loss of viability in the wt strain was found, which was not observed in the mutant cells (Figure 3C, lower panel). We performed a similar experiment using streptomycin (Sm), another protein synthesis inhibitor, at 20 µg.ml^−1^ (selection for transformants to Sm-resistance is 100 µg.ml^−1^). In this case, we observed, again, that the mutant cells were more tolerant to Sm-treatment than the wild type strain (Figure S2). We conclude that the *relBE2Spn* operon seems to be activated when protein synthesis is inhibited, so that under these unfavourable conditions this TAS could contribute to modulate the survival response through stasis.

### The *relBE2Spn* locus is conserved among *S. pneumoniae* clinical isolates

**Figure 4:**
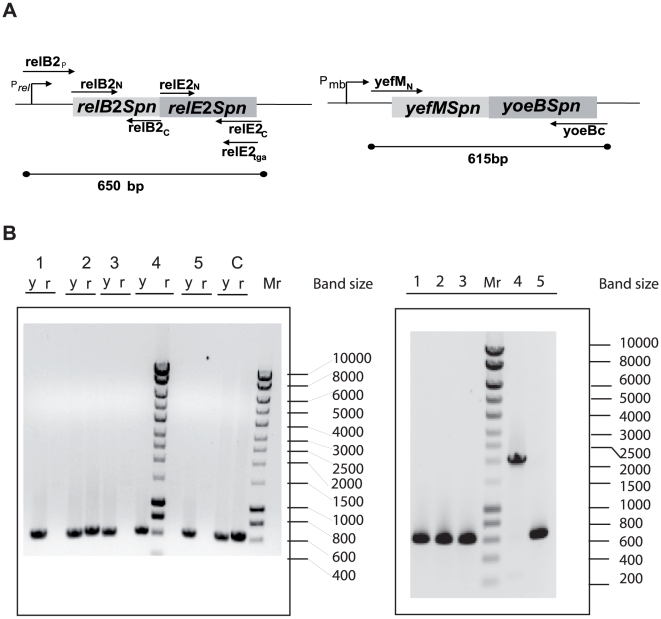
Presence of the *reBE2Spn* operon in the chromosome of isolates of *S. pneumoniae*. Genetic organization of the *relBE2Spn* and *yefM-yoeBSpn* loci in the R6 strain; the position of promoters P_rel_ and P_mb_, respectively, are shown (A). The primers used in the PCR amplifications are drawn as arrows, and the expected sizes of the corresponding PCR fragments are shown below. The PCR products detected using the oligonucleotide pairs relB2_p_ /relE2_c_ and yefM_N_ /yoeB_C_ (left panel) or the oligonucleotide pair relB2_p_/relE2tga (right panel) were separated on agarose gels (B). DNAs from loci *relBE2Spn* (r) or from *yefM-yoeBSpn* (y) were isolated from different *S. pneumoniae* macrolide-resistant strains, as follows: CipR-67 (1); CipR-25 (2); CipR-22 (3); CipR-14 (4); CipR-23 (5); R6wt (C); Mr. Molecular weight standard, Hyper ladder I (BIOLINE).

We reasoned that if the expression of *relBE2Spn* could confer a selective advantage to the pneumococcus, then a conservation of this locus in the bacterial chromosome of most, if not all, isolates should be expected, in spite of hyper-recombination typical for this species [45]. A preliminary analysis was performed in a small set of five Spanish clinical isolates, for which the presence of the *relBE2Spn* and the *yefM-yoeBSpn* loci (another pneumococcal TAS that was used as a control), was tested by PCR using the oligonucleotide pairs relB2_p_/relE2_c_ and yefM_N_/yoeB_C_, respectively. The first pair was previously used to amplify the *relBE2Spn* locus of R6 strain [33], the relE2_c_ primer annealing partially into the region encoding RelE2Spn toxin (Figure 4A). In these five strains, amplification of the *yefM-yoeBSpn* locus was feasible, in contrast to *relBE2Spn* (Figure 4B, left panel). However, when oligonucleotide relE2tga (fully complementary to the 3′ region encoding the toxin gene) was used instead of primer relE2_C_, a PCR product was detected (Figure 4B, right panel). The size of one of the PCR products was bigger than expected (around 2000 bp instead 650 bp), due to the presence of the IS1167 sequence (see below). The results obtained for the Spanish isolates demonstrated that the five strains analyzed contained the *relBE2Spn* locus and all but one (strain CipR-25) exhibited changes compared to strain R6 in the chromosomal regions flanking the *relBE2Spn* operon, whereas the region around the *yefM-yoeBSpn* locus was kept intact.

**Figure 5:**
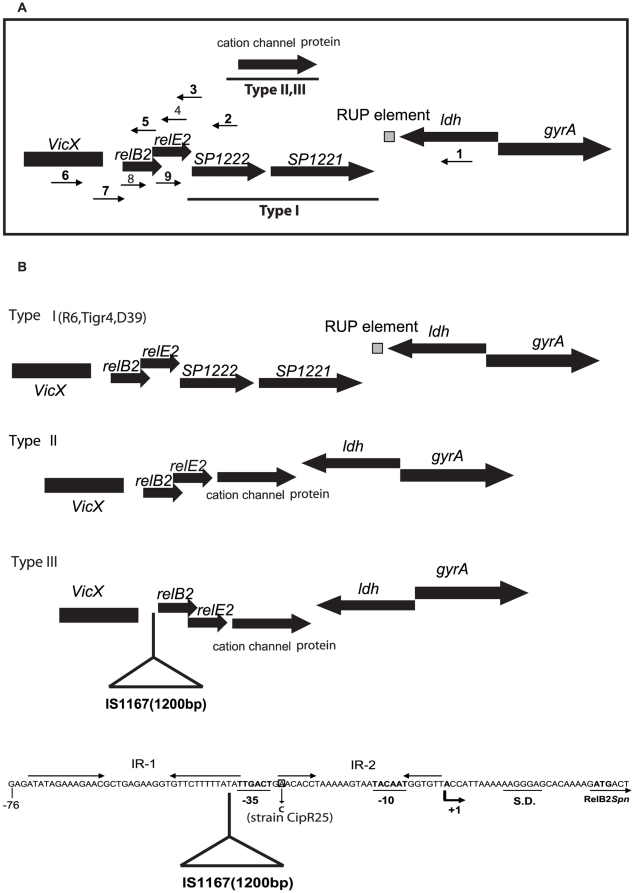
Polymorphisms found at the *relBE2Spn* locus in clinical isolates of *S. pneumoniae*. A genetic diagram of the chromosomal region flanking the *relBE2Spn* locus in the R6 strain shows that this region includes genes encoding Ldh (lactate dehydrogenase), GyrA (the A subunit of DNA gyrase), and SP1221 and SP1222 (putative type II restriction endonucleases). These orfs have been detected only in the pneumococcal strains belonging to Type I, whereas the putative cation channel protein was found in those strains included in both Type II and Type III. Other genes identified in this region were: *relE2* (*relBE2Spn* locus encoding toxin RelE2), *relB2* (*relBE2Spn* locus encoding antitoxin RelB2), and *vicX* (encoding VicX protein). The *vicx* gene comprises part of the TCS02 operon (vicRKX) essential for pneumococcal viability. In addition, a RUP element has been identified between the *ldh* and the *sp1221* coding regions. RUP elements are an insertion sequence (IS)-derivative that could still be mobile [62]. The collection of oligonucleotides used are also indicated by a number corresponding to the following oligonucleotides: **1**, ldh_ter,_; **2**, SP1222; **3**, relE2_C_; **4**, relE2_tga_; **5**, relB2_C_; **6**, rel2p5′; **7**, relB2p; **8**, relB2_N_, **9**, relE2_N_. The genetic organization of the three types of *relBE2Spn* loci is depicted (A). Illustration of the three types of *relBE2Spn* locus genetic organization found out in a collection of *S. pneumoniae* clinical isolates (B) At the bottom, the region spanning the *relBE2Spn* promoter [33] is depicted. The location of the IS1167 transposon and the mutation identified in strain 1531 (CipR-25) are highlighted.

These initial polymorphisms prompted us to perform a similar search in a variety of clinical isolates. To this end, we chose more strains from well-characterized collections of clinical isolates from Spain and Poland [46]–[48]. The Spanish collection (Table S1) consisted of 12 more isolates whose serotypes and majority of sequence types (STs) had been characterized, with the exception of four isolates for which STs were established in this study (Table S1). Apart from its role in epidemiology, multi-locus sequence typing (MLST) provides genetic information of the population structure [45]. MLST is performed by comparison of the DNA sequences of internal fragments of seven housekeeping genes of an isolate with these available at the MLST database (http://spneumoniae.mlst.net/). Spanish strains were isolated from blood and sputum in the years 2002 and 2006. The Polish clinical isolates proceeded from the National Medicines Institute pneumococcal collection, and amounted to 58 isolates representing 37 serotypes and 52 different STs [48]. These strains were mainly isolated from cerebral spinal fluid during the years 1997 to 2002. Spanish and Polish isolates were tested for presence of the *relBE2Spn* locus and its flanking regions by amplification with different oligonucleotides spanning the appropriate regions (Figure 5A). In addition to those, we checked, through bio-informatics procedures, the presence of the *relBE2Spn* operon in another 31 strains whose sequences are available at the NCBI Genome Project (http://www.ncbi.nlm.nih.gov/) and at the Sanger Institute (http://www.sanger.ac.uk/Projects/S_pneumoniae/), thus making a total of 100 strains analyzed (Table S1). In addition, STs of sequenced pneumococcal strains for which MLST data was not available were determined *in silico*.

The results of the global PCR analyses of the chromosomal structure of *S. pneumoniae* around the *relBE2Spn* locus allowed us to classify the 100 isolates tested into three categories (Figure 5B and Table S1): Type I shared the genetic organization found in known strains (TIGR4, D39, and R6). Type II lacked the open reading frames (orf) SP1222 and SP1221, which were replaced by an orf homologous with the cation channel protein family. The genetic organization of Type III was similar to Type II but, in addition, it contained a 1200 bp sequence insertion located upstream the promoter of the *relBE2Spn* operon. The nucleotide sequence of this insertion corresponded to the IS1167 transposon sequence, which includes inverted repeats flanking the transposase [49]. There was no association of the TAS types with pneumococcal serotypes. However, a very good concordance was observed in all cases when more than a single representative of a given ST was analyzed. Altogether, the latter observation was made for 15 STs that included 44 isolates. Determination of the nucleotide sequence of several of the clinical isolates belonging to the three pneumococcal *relBE* Types (Table S1) showed no sequence changes in the antitoxin-encoding *relB2Spn* gene. However, in the region encoding the RelE2*Spn* toxin, several nucleotide changes were detected. Some of them corresponded to silent mutations (GTC to GTT at V74; ATC to ATT at I65; GAC to GAT at D39, and TGA to TAA at the stop codon of the gene). Two other polymorphisms in which minor amino acid changes occurred were also found (T34I, and D39G), whereas some strains, like CipR-25 (Figure 5B), exhibited changes in the region spanning the -35 and -10 region of the *relBE2Spn* promoter, a region probably involved in the transcriptional self-regulation of the operon (I. Moreno, C. Nieto and M. Espinosa, unpublished).

Even though the nucleotide sequence of the *relBE2Spn* promoter region in the different types was not essentially modified, synthesis of the *relBE2Spn* mRNA (and hence the expression of these two proteins) could be affected. To detect the *relBE2Spn* mRNA in several clinical isolates, primer extension analyses were carried out. Total RNA was isolated from selected strains belonging to the three genomic types: i) from Type I, strains R6, and CipR-25, the latter containing the A/G change at the position -28 in the *relBE2Spn* proposed regulatory region; ii) from Type II, strains CipR-31, CipR-67, and 2115, the latter harbouring also the same change in the proposed regulatory region, and iii) from Type III, CipR-51 and CipR-14. In all strains, a primer extension product was detected (Figure S3) and its size was the same as the one detected previously for the laboratory R6 strain [33].

Taken together, we can conclude that all strains analyzed retained the *relBE2Spn* module, but exhibited three different genetic arrangements: 21.5%, 61.3% and 17.2% of the analyzed strains exhibited a genetic organization of the type I, II and III, respectively; 36% of the sequenced strains bore mutations in the gene encoding the RelE2*Spn* toxin. Furthermore, the operon organization, and thus co-transcription of both genes, was maintained in all strains tested.

### Attenuated toxicity in *relE2Spn* mutants

**Figure 6:**
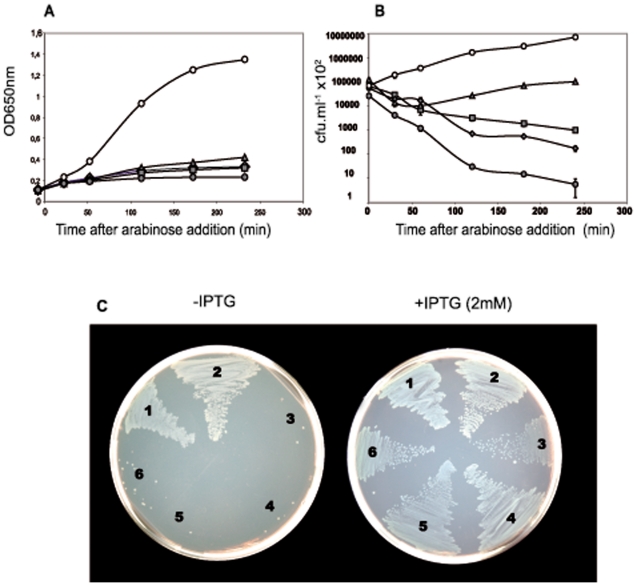
Functional analysis of RelE2*Spn* mutants in *E. coli*. Cell growth arrest subsequent to the expression of the *relE2Spn* wt gene or the mutants T34I or D39G *E. coli* TOP-10 cells harbouring plasmids pE2wt (•), pE71(D39G mutant) (▴), pE81(T34I mutant) (♦), pE600 (T34I mutant) (▪) or pEter as a control (o) were exponentially grown in TY medium containing 0.4% glucose and Km to an OD_600_  = 0.15. Then, cultures received 0.4% arabinose and growth was measured by determination of the OD_600_ (A) and by counting the cfu (B), this latter by plating appropriate dilutions on medium supplemented with 0.4% glucose and Km and incubated overnight at 37°C. All the experiments were performed at least in duplicate. The effect of the separate and of combined expression of the *relE2Spn* or *relB2Spn* in *E. coli* was also tested on solid medium (C). *E. coli* TOP-10 cells harbouring pB2wt and pEter (streak 1), pFSU2 (streak 2), pE2wt (streak 3), pE71 (streak 4); pE81 (streak 5); pE600 (streak 6) were streaked on TY plates containing Km and Ap, supplemented with 0.4% arabinose and with or without IPTG (2 mM). Arabinose induces the toxin expression, whereas IPTG (2 mM) promotes the antitoxin overproduction.

As shown above, most of the nucleotide changes identified in the sequence of the *relBE2Spn* locus were located within the *relE2Spn* gene, two of them being missense mutations that affected the RelE2*Spn* toxin: change D39G was found in three strains (Polish 7153, and the NCBI genome project P1031 and JJA), whereas change T34I was relatively frequent (around 35% of the sequenced isolates). To verify whether these changes affected the toxic activity of RelE2*Spn*, and to elucidate their possible physiological consequences, we tested toxicity on *E. coli* cells based on two criteria previously used for the pneumococcal *yefM-yoeB* operon [34]: i) inhibition of cell growth after expression of either wild type or mutant RelE2*Spn*, and ii) ability of RelE2*Spn* to interact with the cognate RelB2*Spn* antitoxin in cultures harbouring uncoupled genes (*relB2Spn* and *relE2Spn*) cloned in two different plasmids under inducible promoters. In the latter conditions, induction of the antitoxin should neutralize the toxic effect of RelE2*Spn* thus permitting bacterial growth. To this end, DNA fragments containing genes *relE2Spn*, wt or mutants harbouring the D39G, T34I, and, as a control, *relE2ter* (encoding a truncated and inactive RelE2*Spn* protein) were cloned into plasmid pFUS2 [50] under the control of the *araBAD* promoter (P_BAD_), which is inducible by arabinose and repressed by glucose. The resulting plasmids (Table 1) were termed pE2wt (wt RelE2*Spn*), pE71 (D39G RelE2*Spn*), pE81 (from strain 8651) or pE600 (from strain k-600), both harbouring the T34I RelE2*Spn* mutation and pE2ter (truncated RelE2 protein). In addition, the *relB2Spn* wt gene was cloned in plasmid pNM220 [51], which allows IPTG-inducible expression of the antitoxin from the P_lac_ promoter; the resulting plasmid was termed pB2wt (Table 1). As expected, no significant difference in growth rate was observed for *E. coli* cells with the control pE2ter upon induction of P_BAD_. However, a total growth arrest was observed for *E. coli* harbouring the plasmids pE2wt, pE71(D39G), pE81(T34I), or pE600(T34I) (Figure 6A). Additionally, a severe decrease in the number of viable cells compared to cultures containing the pE2ter plasmid was seen (Figure 6B). This reduction in cfu occurred in the following order: *relE2*wt (almost four orders of magnitude) > *relE2*T34I (nearly two orders) > *relE2*D39G (twofold). The toxicity of the RelE2*Spn* toxin could be counteracted by its cognate antitoxin, encoded in the pB2wt plasmid. Cells, containing the different pairs of plasmids were streaked on plates supplemented with 0.4% arabinose (induction of toxin synthesis) with or without 2 mM IPTG (induction of antitoxin synhtesis). Transformants containing, in addition to pB2wt, plasmids encoding the toxin (totally or partially functional) were able to grow only on plates supplemented with IPTG while control cells harbouring pB2wt and either pFUS or pE2ter did not show growth differences in the presence or absence of IPTG (Figure 6C).

## Discussion

The human upper respiratory tract is a natural environment for *S. pneumoniae* from which these bacteria spread to other body parts and to new hosts; hence an increase in pneumococcal persistence during colonization may influence its virulence and epidemicity [52]. Persistence may be one of the roles performed by the bacterial TAS by allowing bacteria to survive under nutrient limited conditions, thereby improving adaptability to selective pressures and permitting the bacteria to retain their capacity to colonize humans without reduction in virulence. The pneumococcal *relBE2Spn* was identified in the chromosome of *S. pneumoniae* and shown to be functional, in contrast to the pneumococcal homologue termed *relBE1Spn* [16], [33]. Cells lacking the *relBE2Spn* operon exhibited the same growth profile and response to sugar starvation as the wt bacteria did (Figure S1). Differences were found, however, when cells were subjected to protein synthesis inhibition either by amino acid starvation or antibiotic treatment. In the case of the *E. coli* RelBE, the toxin was activated because of antitoxin RelB degradation subsequent to inhibition of protein synthesis [39]. Toxicity of *Eco*RelE protein is due to cleavage of translating mRNA at the ribosomal A site [12], [53]; other RelE homologues, including RelE2*Spn* seem to cleave RNA in a similar manner, and thus cells exposed to the toxin showed a drastic growth arrest [16], [33]. Similarly, activation of *RelE2Spn* by SHT treatment, led to reduction in the number of cfu (Figures 1 and 2), but this reduction was not due to cell death as detected by the LIVE/DEAD BacLight bacterial viability method (not shown), but rather to a slower rate of cell growth. After SHT removal, cells returned to normal growth, although two major differences were observed between the wt and the mutant strains (Figures 1 and 2): i) the wt cells recovered more slowly than the mutants, most likely because recovery of cell growth of the former required prior antitoxin synthesis to neutralize the RelE2*Spn* toxin, and ii) the wt strain exhibited an exponential growth period after recovery which was longer than that of the mutant. These results indicated that the pneumococcal *relBE2* system, under amino acid starvation, could help the bacteria to divert the scarce resources to essential processes, thus improving its survival potential. Treatment with Erm (or with Sm) also resulted in a different response in the two pneumococcal strains used. The wt showed a higher and quicker reduction in cfu than the mutant (Figures 3B and S2) although, at 0.1 µg ml^−1^ of Erm, it was not due to diminished cell viability. However, at 1 µg ml^−1^ of Erm, a clear loss in viability was observed for the wt, which was not the case for the mutant (Figure 3C). The higher sensitivity of the wt strain to a low dosage of Erm can be explained as the result of RelE2*Spn* activation subsequent to protein synthesis inhibition. When higher concentrations of the antibiotic were used, cell lysis was observed in the wt after 180 minutes of treatment and, during the recovery period, only the mutant cells were able to resume growth but only after 24 hours of Erm removal (not shown).Thus, activation of the pneumococcal *relBE2Spn* operon subsequent to antibiotic treatment appeared to induce an extreme interruption of the protein synthesis, leaving the bacteria unable to recover viability or even inducing cell death. Then, lack of the *relBE2Spn* operon in *S. pneumoniae* would lead to antibiotic tolerance a role that coincides with the one proposed for the *E. coli mazEF* TAS [21].

The pneumococcal *relBE2Spn* operon is not essential, at least under the laboratory growth conditions used ([33] and Figure S1), but it showed a functional conservation in all the strains tested (see below). This was unlike the two other TAS characterized in *S. pneumoniae*, namely *pezAT* and *yoeB-yefMSpn*. The former was found to be absent in several clinical isolates of *S. pneumoniae* [7], whereas a search for the presence of *yoeB-yefMSpn* in 31 pneumococcal strains sequenced (NCBI project or Sanger institute) showed that more than 40% of them lacked this TAS (not shown). In *E. coli*, the homologous *relEB* TAS have been lost in several strains [14]. In addition, analyses of 395 *E. coli* strains showed decay in the chromosomally-encoded *ccdAB* TAS and a molecular evolution analysis of these data suggests that this TAS does not seem to retain any role in *E. coli* [54]. A recent study on comparative metagenomic analyses of plasmid-encoded functions in the human gut microbiome showed that the RelBE TAS, as compared to other TAS, is relatively abundant and retains a broad phylogenetic distribution in the human gut microbiome, suggesting that prevalence of RelBE could be related to fitness of the bacterial host [55]. Our analysis of the *relBE2Spn* locus showed the existence of three different genetic organizations, although transcription of the operon was not affected by these rearrangements (Figure S3). Type II seems to be most divergent and most prevalent (Table S1). This may suggest this is an ancestral type. It is easy to imagine how types I and type III are made by single genetic events from II. Type III looks homogenous but all the isolates of type III that were sequenced originated from the same clone so this could have been expected.

**Figure 7:**
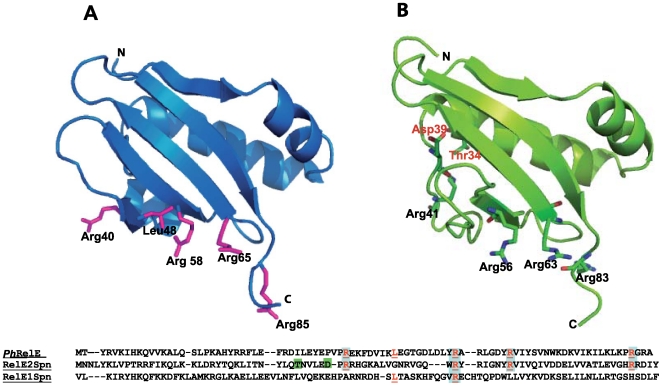
Molecular modelling of RelBE2*Spn*. Structure of RelE from *P. hirokoshii* (*Ph*RelE) (A) and structural model obtained for the pneumococcal toxin RelE2*Spn* (B). Residues important for protein synthesis inhibitory activity of *Ph*RelE are displayed. In the case of RelE2*Spn* model, the Arg residues which aligned with those of *Ph*RelE involved in protein synthesis inhibition are shown. Residues changed in the RelE2 pneumococcal mutants isolated here, T34 and D39, are indicated in red. The sequence alignment of *Ph*RelE, RelE2*Spn* and RelE1*Spn* (a pneumococcal non-toxic homolog) is shown in the bottom of the figure. Residues in the *Ph*RelE, involved in toxin activity are red underlined and the corresponding residues in RelE1*Spn* and RelE2*Spn* are highlighted in the same way. T34 and D39 positions in RelE2*Spn* are framed in green.

In addition to the above arrangements, various isolates showed several more polymorphisms at the *relE2Spn* gene, some of them affecting the amino acid sequence of the protein. Two of them were found to be relevant, namely changes T34I, and D39G, since toxin activation in either of the two mutants led to growth arrest, although their toxic effect was lower than the wt toxin (Figure 6), indicating that these amino acid changes could affect critical residues of the toxin. We constructed a molecular model of the pneumococcal RelE*Spn* based on the crystal structures of the RelBE protein complexes of *P. horikoshii* (*Ph*RelBE) [10] and the recently published structure of the RelE protein from *Methanococcus jannaschii* (*Mj*RelBE) [11]. Amino acid sequence alignment and the structural model (Figure 7) indicated that all R residues that were previously related to *Ph*RelE toxicity (R40, R58, R65, and R85) were conserved in RelE2*Spn* (R41, R56, R63, and R83). Curiously, this R-distribution was not fully conserved in the pneumococcal RelE1*Spn* (also present in the R6 strain; Figure 7) perhaps causing its lack of functionality [33]. In the RelE2*Spn* molecular model (Figure 7), residues T34 and D39 (changed in the RelE2*Spn* low toxicity mutants to I and G, respectively) appeared to be located close to the toxicity-related R residues. According to the structure of toxins Kid and MazF [56]–[58], we can postulate that: i) mutation T34I would allow the toxin to retain its RNase activity but with slight changes in its substrate binding capacity, and ii) D39, together with E38, are acidic residues that could act in the catalysis of RNA in the active site of the toxin so that mutation D39G would reduce the RelE2*Spn* mRNA cleavage activity, thereby diminishing RelE2*Spn* toxicity (Figure 6). The model of RelE2*Spn* also sheds light on some possible structural peculiarities of the toxin: unlike its *E. coli* and *P. horikoshii* homologues, RelE2*Spn* would include in its catalytic site (besides the conserved R residues), residues H43, Y31 and Y57, and two acidic residues (E38 and D39) (Figure 7 and Figure S4). These residues are present in the catalytic site of toxins with ribosome-independent RNase activity, such as YoeB [6], Kid [57] and MazF [58]. The presence of these additional residues allow us to speculate that RelE2*Spn* could mediate the cleavage of translating mRNA [16] but also could have an intrinsic RNase activity able to cleave untranslated mRNA, as shown for *Ecoli*YoeB [6].

### Concluding remarks

Our results indicate that the *relBE2Spn* locus could provide a mechanism for *S. pneumoniae* to cope with unfavourable conditions, allowing the bacteria to efficiently survive and colonize humans. Further, the results show the importance of TAS as targets for designing new antimicrobials, which is especially true for bacteria like *S. pneumoniae* because of their high recombination rates and, being naturally competent, horizontal transfer. As a consequence, the appearance of new polymorphic and antibiotic resistant strains poses a serious threat for infection management.

## Materials and Methods

### Bacterial strains, growth conditions and plasmid constructions

Strains and plasmids used in this study are listed in Table 1. *E. coli* cultures were grown in TY medium [59] with selection for ampicillin resistance (Amp^R^, 150 µg.ml^−1^), or kanamycin resistance (Km^R^, 50 µg.ml^−1^). *S. pneumoniae* cells were grown in AGCH medium [40] supplemented with 0.3% sucrose and 0.2% yeast extract (complete AGCH) medium, with or without selection for resistance to chloramphenicol (Cm^R^, 2 µg. ml^−1^). All cultures were grown at 37°C. The agar plates were incubated at 37°C in air. SHT (used at 1.5 mg.ml^−1^; a compound which specifically blocks charging of seryl-tRNA, thus inhibiting protein synthesis), Erm (used at 0.1 or 1 µg. ml^−1^), and Sm (used at 20 µg.ml^−1^) both of them blocking bacterial ribosomes, were purchased from Sigma. We used Erm instead Cm because the mutant strain, R6*ΔrelB2Spn*, harbours a chromosomally-integrated copy of the *cat* gene (Table 1).

Plasmids used in this work were constructed as follows: pE2wt, pE71, pE81, and pE600: The *relE2Spn* gene with its own ribosome binding site was amplified by PCR from chromosomal DNAs of strains R6 (pE2wt), 7153 (pE71), 8151 (pE81), or K-600 (pE600) and amplified with primers relE2_N_ (5′-CGCG GATCCGATGCATGATTTAGGCTTGAAGGATGAATA-3′) and relE2_tga_ (5′-CGTGGTACCTCAATAAATATCTCTCCGATGACCAACTTC-3′). The resulting 290-bp PCR products were digested with *Eco*RI and *Kpn*I before ligation into the equivalent sites of pFUS2. Plasmid pE2ter was randomly isolated during construction of pE71 and contains a mutation in the *relE2*sequence changing the E38 residue for a termination codon, yielding a truncated RelE2 protein. Plasmid pB2wt was constructed by amplification of a chromosomal DNA fragment encoding gene *relB2Spn* with its own ribosome binding site using the primers relB2_BamHI_ (5′-CGGGATCCGTGTTACCATTAAAAAAGGGAGCACA AAG-3′) and relLC_C_ (5′-CGGGGTACCATCGCGAATTCTAAAACGTCTTGTT GGAACTAATTTATAC-3′). The resulting 310-bp DNA fragment was digested with *Bam*HI and *Eco*RI before ligation into the equivalent sites of pNM220. All plasmids were rescued by transformation of competent *E. coli* cells.

### Growth and recovery tests in *S. pneumoniae* cells

Cultures of *S. pneumoniae* R6 and R6*ΔrelB2Spn* were exponentially grown in AGCH complete medium to OD_650_ = 0.2 at 37°C. Then, half of each culture was exposed to the different experimental conditions: carbon starvation, or addition of SHT or Erm (or Sm). Growth of treated and untreated cultures was followed by OD_650_ and the viability (number of cfu) was measured by plating serial dilutions of each culture on AGCH plates. After SHT-, Erm, or Sm-treatment, cultures were washed twice with pre-warmed AGCH and suspended in the same volume of complete AGCH drug-free medium. OD_650_ and viability was then tested for at least 180 minutes. In the carbon starvation experiments exponentially growing cultures (to OD_650_ = 0.2) were washed twice in pre-warmed AGCH medium and finally suspended in the same volume of AGCH medium supplemented with 0.2% yeast extract and with or without sucrose. Cell growth and viability were measured as above. All the experiments were performed at least three times.

### Fluorescence microscopy

Cultures of R6 and R6*ΔrelB2Spn* were exponentially grown at OD_650_  = 0.25 in AGCH complete medium at 37°C and Erm was added at two concentrations (0.1 µg.ml^−1^ or 1 µg.ml^−1^). After 180 min, cells were harvested by centrifugation, washed twice with buffer (50 mM Tris-HCl pH 7.6, 100 mM NaCl, 8 mM MgSO_4_) and stained with the LIVE/DEAD BacLight Bacterial viability kit (Invitrogen) according to the manufacturer's instructions. Cells were visualized using a multidimensional AF6000 LX LEICA microscope and filter cube L5 for green fluorescence or N5 to detect red fluorescence.

### PCR-based gene detection in *S. pneumoniae* and MLST

Chromosomal DNAs were extracted using the Bacterial Genomic DNA Isolation Kit (NORGEN) and chromosomal DNA (50–100 ng) was added to PCR reactions performed using Phusion high fidelity DNA polymerase (Finnzymes) and, as primers, the following oligonucleotides:

yefM_N_: 5′-CGCGGATCCGCTTGTACAAGTTCCTGACAATTTC-3′

yoeB_C_: 5′-CTGGAATTCCGGTAGAGACTTGAGAAAAAGCCTA-3′

rel2p5′: 5′-CGGAATTCCGATCAGGTTCTTACGCTTGGCG-3′

relB2p: 5′-CAGATAC CGCAACACCATTGACAG-3′

relB2_N_: 5′-TGCTCCCGGGCTATTACATTAAAAGTTTCTGAA GCTG-3′

relB2_C_: 5′-CGCGAATTCCTTCCCAAGTAATGGGT TCAACTCC-3′

relE2_N_: 5′-CGCGGATCCGATGCATGATTTAGGCTTGAAGGATGAATA-3′

relE2_tga_: 5′-CGTGGTACCTCAATAAATATCTCTCCGATGACCAACTTC-3′

SP1222: 5′-CCTCACGACTAATCCGTTGCAG-3′

ldh_ter_: 5′-GCATCTGCTAA AGAATTACAAGCATCATTG-3′

To analyse the IS1167 transposon sequence, a 1750-bp PCR-DNA fragment including this element was obtained using as template chromosomal DNA isolated from strain 2167 and, as primers, relB2p and relB2_C_. The sequence of this DNA fragment was determined using primers relB2p, relB2_C_, and two specific IS1167 primers: 2167_N_ (5′-GTCATAGTAAGGACTAAACATA TCC-3′) and 2167_C_ (5′-GAAAAGCGATCAAACAACTCATTAG-3′).

MLST based on sequencing of fragments of seven housekeeping genes, *aroE, gdh, gki, recP, spi, xpt* and *ddl* was performed as described by others [60]. The database http://spneumoniae.mlst.net/was used to assign allele numbers and STs.

### Primer extension analysis

Total RNA was isolated from R6 and from different pneumococcal isolates with Aurum total RNA minikit (BIO-RAD). For RNA extraction, 1.5–3 ml of the bacterial cultures in late exponential phase were centrifuged and the cells were resuspended in 100 µl of lysis buffer (50 m M Tris-HCl pH 7.6, 1 mM EDTA, 50 mM NaCl, 0.1% sodium deoxycholate). The cell suspension was incubated 10 min at 30°C and further preparation was done according to the manufacturer's instructions. Primer extension assays were performed as described [61] using either a radiolabelled *relBE2Spn* specific primer (*rel*RNA, 5′-GAAACTCCTTCAAACTTAGCC-3′) [33] or a *malX* specific primer mal1, 5′-GTGTAACAGTTCCAAGCACCG-3′). The 3′-ends of primers were located 56 nt or 48 nt from the nucleotide A of the ATG initiation codon of the *relB2Spn* gene or in the *malX* ATG initiation codon, respectively [61].

### Model construction

The three-dimensional model of RelE2*Spn* toxin was constructed using Geno3D molecular modelling program (pBIL.icp.fr.Geno3D, http://geno3d-pbil.ibcp.fr) and the 2.3 Å resolution X-ray crystallographic structure of the *P. horikoshii* OT3 aRelBE complex [10] (PDB ID:1wmi), and the 2.1 Å resolution X-ray crystallographic structure of the *M. jannaschii Mj*RelBE complex [11], (PDB ID:3BPQ) as templates. The graphic display was performed with PyMOL program (DeLano Scientific LLC, http://www.pymol.org).

### Web sites

NCBI Genome Project: http://www.ncbi.nlm.nih.gov/

Pneumococcal MLST database: http://spneumoniae.mlst.net/

European Committee on Antimicrobial Susceptibility Testing: http://www.eucast.org/mic_distributions/

PyMOL: http://www.pymol.org.

Sanger Institute: http://www.sanger.ac.uk/Projects/S_pneumoniae/

Geno 3D: http://geno3d-pbil.ibcp.fr

## Supporting Information

Figure S1 Growth profile of S. pneumoniae cells wt and mutant R6(capital delta)relB2Spn under normal or carbon-starvation conditions. Pneumococcal strains R6 (triangles) or (capital delta)relB2Spn (circles) were grown in AGCH complete medium (A, B). In carbon-starvation conditions (panels C and D), cells were exponentially grown in AGCH complete medium to an OD650  = 0.2, twice washed, and suspended in the same medium with (open symbols) or without sucrose (filled symbols). Growth of the cultures was followed by measurement of OD650 nm (A, C) or by determination of the number of cfu (B, D).
(1.35 MB EPS)

Figure S2 Inhibition of protein synthesis mediated by Sm treatment. *S. pneumoniae* cells from strains R6 (circles) or R6(capital delta)relB2Spn (triangles) were grown exponentially in complete AGCH medium to an OD650  = 0.1−0.14. Then, Sm (20 (mu)g.ml^−1^) was added, and incubation was continued for 180 min more. Growth was followed by measurement of OD650 nm of the cultures untreated (open symbols) or treated (closed symbols) with Sm (A). At indicated times appropriate dilutions of cells were plated and incubated as in Figure 1 (B).
(1.11 MB EPS)

Figure S3 Primer extension analysis using total RNA from different S. pneumoniae clinical isolates. RNA samples from R6 (1) and the following clinical isolates: CipR25 (2), 2115 (3), CipR67 (4), CipR31 (5); CipR14 (6), and CipR51 (7) were annealed with [32P]-labelled specific primers mal1 (x; as a control) or with the relRNA oligonucleotide (r) to detect relBE2Spn mRNA. Ct Indicates a G+A Maxam and Gilbert sequencing reaction, used as DNA size marker.
(0.53 MB EPS)

Figure S4 RelE2Spn three dimensional structural model. Location of R41, R56, R63, R83, and D39 residues is depicted. Other residues (Y31 and Y57, H43 and E38) supposedly involved in the catalytic mechanism are displayed in cyan.
(1.06 MB EPS)

Table S1 (0.04 MB XLS)
